# A minimal ion–chemistry model for predicting benign paroxysmal positional vertigo risk based on endolymphatic calcium and pH

**DOI:** 10.3389/fneur.2025.1690931

**Published:** 2025-10-23

**Authors:** Dong-Gyun Han

**Affiliations:** Dr Han's Neurology Clinic, Dong-gu, Daejeon, Republic of Korea

**Keywords:** benign paroxysmal positional vertigo, otoconia, Ionized calcium, predictive modeling, carbonate chemistry, computational simulation

## Abstract

Benign paroxysmal positional vertigo (BPPV) arises from detachment of otoconia—calcium carbonate (CaCO_3_) crystals embedded in a protein matrix—whose stability depends on endolymph ionic composition and *pH*. Age-related calcium metabolism, acid–base imbalance, and hormonal factors can impair otoconia integrity, yet, to our knowledge, no prior quantitative model integrates these biochemical parameters to predict BPPV risk. Beyond the established mechanical mechanisms of canalithiasis and cupulolithiasis, we introduce a parsimonious biochemical model in which the endolymphatic saturation index (Ω), governed by *pH* and ionized calcium [*Ca*^2+^], delineates an otoconia stability–dissolution boundary (Ω≈1) and complements the mechanical framework. Using carbonate-equilibrium chemistry and the CaCO_3_ solubility product (*K*_*sp*_), we compute Ω and derive the critical calcium concentration *C*_*crit*_(*pH*) at Ω = 1. A logistic mapping of $\text{Δ}C=C_{crit}\left(pH\right)-\left[Ca^{2+}\right]$ yields a dimensionless relative-risk score. Systemic and environmental states are represented as shifts in *pH* and [*Ca*^2+^], and a synthetic cohort (*N* = 10,000) visualizes *pH*[*Ca*^2+^] risk contours and the Ω = 1 boundary. States with Ω>1 (supersaturation) predict otoconia stability, whereas Ω < 1 (undersaturation) predicts dissolution; hyperventilation and thiazide diuretics tend to increase Ω, while metabolic acidosis, hypoventilation, and loop diuretics reduce it; acetazolamide (carbonic-anhydrase inhibition) typically induces metabolic acidosis and therefore lowers Ω. The combination of low *pH* and reduced [*Ca*^2+^] markedly expands the Ω < 1 dissolution-prone domain, with the Ω = 1 contour acting as a dynamic equilibrium sensitive to small biochemical changes. In simulations, the risk distribution was right-skewed (mean *R*≈0.78; 80% with *R*>0.68). Because direct endolymph sampling is impractical, we propose serum ionized calcium together with blood-gas–derived $pH/HCO_{3}^{-}/pCO_{2}$ as non-invasive surrogates for relative-risk inference (a blood-based Ω proxy), not one-to-one estimators of absolute vestibular chemistry. This deterministic, two-input minimal framework is hypothesis-generating and complementary to the mechanical model; prospective, surrogate-based calibration and robustness testing (to *C*_*T*_, ionic strength/activity coefficients, *K*_*sp*_, and temperature) are required before clinical use.

## 1 Introduction

Benign paroxysmal positional vertigo (BPPV) is the most common peripheral vestibular disorder, with a lifetime prevalence of ~2.4% (1). It results from displacement of otoconia from the utricular macula into the semicircular canals (2, 3). Otoconia are biomineralized structures composed mainly of calcium-carbonate (CaCO_3_) crystals embedded in an organic (proteinaceous) matrix (4, 5). In contrast to bone mineral—largely hydroxyapatite *Ca*_10_(*P*_*O*_4_)6_(*OH*)_2_, a calcium phosphate with a highly stable lattice and strong ionic bonding—calcite CaCO_3_ exhibits weaker ionic bonding and greater chemical reactivity. This lower intrinsic stability renders otoconia more vulnerable to dissolution under acidic conditions or shifts in ionic composition, whereas hydroxyapatite confers long-term structural integrity to bone.

Otoconia stability is critically modulated by calcium metabolism and endolymph *pH* (6, 7). Age-related reductions in intestinal calcium absorption, estrogen deficiency, and systemic disorders such as osteoporosis, migraine, and Ménière's disease perturb endolymphatic calcium homeostasis (8–12). Endolymph *pH*, governed by systemic respiratory and metabolic acid–base balance, sets carbonate speciation; acidification shifts the equilibrium toward bicarbonate/dissolved CO_2_, increasing CaCO_3_ solubility. Experimental and clinical reports indicate that *pH* reduction—whether due to metabolic acidosis, hypoventilation, or local inflammation—destabilizes otoconia and increases the likelihood of BPPV episodes (12, 13).

Prior models have emphasized biomechanical factors, otolithic-membrane integrity, and systemic comorbidities (14, 15), but none have quantitatively linked the biochemical determinants—endolymphatic free calcium [*Ca*^2+^]and *pH* —to BPPV risk. Here, we present a carbonate saturation index–based model in which *pH* and [*Ca*^2+^] jointly determine the stability–dissolution boundary (Ω≈1). This framework complements (rather than replaces) the mechanical paradigm by providing a quantitative map from systemic conditions to predicted otoconia stability and BPPV risk.

## 2 Methods

### 2.1 Model formulation

We quantified otoconia stability using the carbonate saturation index (Ω):


(1) $$\begin{array}{l} \text{Ω}\left(\left[Ca^{2+}\right],pH\right)=\frac{a_{Ca^{2+}+}a_{{CO_{3}}^{2-}}}{K_{sp}}=\frac{\gamma_{Ca^{2+}}\left[Ca^{2+}\right]\gamma_{{CO_{3}}^{2-}}\left[{CO_{3}}^{2-}\right]}{K_{sp}} \end{array}$$

Here, *a* denotes activity (*a*_*i*_ = γ_*i*_[*i*]); γ_*i*_ are activity coefficients (dimensionless) and [*i*] are molar concentrations. Carbonate is obtained from *pH*-dependent speciation as $\left[{CO_{3}}^{2-}\right]=\alpha_{2}\left(pH\right)C_{T}$, where *C*_*T*_ is total inorganic carbon. *K*_*sp*_ is the effective solubility product of calcite CaCO_3_ under the prevailing temperature and ionic-strength conditions. For a minimal model at near-physiological ionic strength (I ≈ 0.15–0.20 M) and 37 °C, we approximate activities by concentrations (γ≡1) and treat *K*_*sp*_ as an effective constant; thus Ω>1 denotes supersaturation favoring precipitation, whereas Ω < 1 denotes undersaturation favoring dissolution. This formulation follows diffusion-controlled CaCO_3_ crystallization (16) and biomineralization studies (17).

Numerator—$\left[Ca^{2+}\right]\left[CO_{3}^{2-}\right]$:

[*Ca*^2+^] is determined by the balance between trans-epithelial calcium influx, efflux, passive diffusion, and protein binding within the vestibular endolymphatic compartment (referring to soluble proteins in endolymph that reversibly bind Ca^2+^, distinct from the structural matrix of otoconia). The $\left[CO_{3}^{2-}\right]$ term is derived from total inorganic carbon (*C*_*T*_) and the *pH*-dependent dissociation fraction α_2_, such that:


(2) $$\begin{array}{l} \left[CO_{3}^{2-}\right]=\alpha 2\left(pH\right)\;C_{T} \end{array}$$

Even small changes in *pH* can produce disproportionately large shifts in $\left[CO_{3}^{2-}\right]$ because α_2_ contains an exponential term, making *pH* a sensitive determinant of the numerator.

Denominator—*K*_*sp*_:

The *K*_*sp*_ term is the solubility product constant for CaCO_3_ at body temperature and ambient pressure, representing the thermodynamic equilibrium condition at which precipitation and dissolution rates are balanced. While temperature and pressure are stable *in vivo*, ionic strength alters ion activities, resulting in a larger effective *K*_*sp*_ when expressed in concentrations. In biological fluids, macromolecules, chelators, and changes in protein composition can also modify the effective *K*_*sp*_ by altering ion activities.

Under stable temperature and pressure, variations in Ω are primarily driven by changes in [*Ca*^2+^], *pH* (via $\left[CO_{3}^{2-}\right]$), *C*_*T*_, and ionic strength.

Carbonate speciation and dissociation fractions:

At 37 °C, carbonate species distribution is determined by the first (*pK*_*a*1_≈6.12) and second (*pK*_*a*2_≈10.3) dissociation constants of carbonic acid, together with the CO_2_ solubility coefficient in plasma (*S*_*C*_*O*__2__≈ 0.0306 mmol L mmHg at 37 °C) (18). Defining:


(3) $$\begin{array}{l} \left[H^{+}\right]=10^{-pH},\;K_{1}=10^{-pK_{a1}},\;K_{2}=10^{-pK_{a2}} \end{array}$$

the normalization constant


(4) $$\begin{array}{l} D={\left[H^{+}\right]}^{2}+K_{1}\left[H^{+}\right]+K_{1}K_{2} \end{array}$$

represents the sum of equilibrium terms for dissolved CO_2_, bicarbonate, and carbonate ions, and serves as the common denominator in the fractional speciation expressions:


(5) $$\begin{array}{l} \alpha_{0}=\frac{{\left[H^{+}\right]}^{2}}{D},\;\alpha_{1}=\frac{K_{1}\left[H^{+}\right]}{D},\;\alpha_{2}=\frac{K_{1}K_{2}}{D} \end{array}$$

where α_0_, α_1_, and α_2_ are the fractions of total inorganic carbon (*C*_*T*_) present as dissolved [*CO*_2_(*aq*)], bicarbonate ions $\left[HCO_{3}^{-}\right]$, and carbonate ions $\left[CO_{3}^{2-}\right]$, respectively. Total inorganic carbon is defined as:


(6) $$\begin{array}{l} C_{T}=\left[CO_{2}\left(aq\right)\right]+\left[HCO_{3}^{-}\right]+\left[CO_{3}^{2-}\right] \end{array}$$

When $\left[HCO_{3}^{-}\right]$ is available (e.g., from blood gas analysis), *C*_*T*_ can be obtained from


(7) $$\begin{array}{l} C_{T}=\frac{\left[HCO_{3}^{-}\right]}{\alpha_{1}} \end{array}$$

#### 2.1.1 Relative risk score (**R**)

The relative risk score was calculated by applying a logistic transformation to the deviation of measured [*Ca*^2+^] from the critical concentration *C*_*crit*_(*pH* ):


(8) $$\begin{array}{l} R=\frac{1}{1\;+\;\exp\left(-\frac{C_{crit}\left(pH\right)-\left[Ca^{2+}\right]}{\sigma_{C}}\right)} \end{array}$$

where σ_*C*_ is a dispersion parameter representing inter-individual variability in tolerance to deviations of [*Ca*^2+^] from *C*_*crit*_. A smaller σ_*C*_ produces a steeper logistic curve, indicating high sensitivity to small departures from *C*_*crit*_, whereas a larger σ_*C*_ yields a shallower curve, reflecting lower acute sensitivity but a greater probability of prolonged residence in destabilizing states. Biologically, σ_*C*_ captures heterogeneity in physiological tolerance, arising from factors such as genetic variation, hormonal status, comorbidities, age, and environmental influences. Empirical estimation of σ_*C*_ can be performed by measuring [*Ca*^2+^] and *pH* in a representative cohort, calculating *C*_*crit*_ for each individual, and fitting the resulting risk distribution to the logistic function–based risk model using regression analysis or maximum likelihood estimation.

#### 2.1.2 Relative critical calcium concentration index

For comparative purposes under constant *K*_*sp*_, we defined a dimensionless index:


(9) $$\begin{array}{l} I_{C_{crit}}\propto {\frac{1}{\alpha 2\left(pH\right)C}}_{T} \end{array}$$

This index allows prediction of relative shifts in *C*_*crit*_ between physiological and pathological states without invasive sampling of endolymph.

Systemic acid–base disturbances measured in blood predictably shift *C*_*crit*_, thereby linking systemic physiology to inner-ear carbonate equilibrium. For example, acidosis (*pH↓*, pCO_2_↑) decreases α_2_, shifting carbonate speciation toward *CO*_2_/${\text{HCO}}_{3}^{-}$ dominance, lowering $\left[CO_{3}^{2-}\right]$, and raising *C*_*crit*_, favoring dissolution (Ω < 1). In contrast, alkalosis increases $\left[CO_{3}^{2-}\right]$ and lowers *C*_*crit*_, promoting supersaturation (Ω>1). Ionic-strength effects computed with Davies activity coefficients (γ) are illustrated in Supplementary Figure S1. These examples highlight, in a qualitative manner, how systemic biochemical states may alter otoconia stability. The framework should be regarded as hypothesis-generating, serving to guide future studies rather than as a validated clinical tool.

#### 2.1.3 Critical calcium at the saturation boundary

We start from the activity-based definition of the saturation index:


$$\begin{array}{l} \text{Ω}=\frac{a_{Ca^{2+}}a_{{CO_{3}}^{2-}}}{K_{sp}}=\frac{\gamma_{Ca^{2+}}\left[Ca^{2+}\right]\gamma_{{CO_{3}}^{2-}}\left[{CO_{3}}^{2-}\right]}{K_{sp}},a_{i}=\gamma_{i}\left[i\right] \end{array}$$

At the precipitation–dissolution boundary (Ω = 1), the critical free calcium is


$$\begin{array}{l} C_{crit}\left(pH\right)\equiv {\left[Ca^{2+}\right]}_{\text{Ω}=1}=\frac{K_{sp}}{\gamma_{Ca^{2+}}\gamma_{{CO_{3}}^{2-}}\left[{CO_{3}}^{2-}\right]} \end{array}$$

Using $\left[{CO_{3}}^{2-}\right]=\alpha_{2}\left(pH\right)C_{T}$ (Equation 2; α_2_ given in Equation 5), we obtain


(10) $$\begin{array}{c} C_{crit}\left(pH\right)=\frac{K_{sp}}{\gamma_{Ca^{2+}}\gamma_{{CO_{3}}^{2-}}\;\alpha_{2}\left(pH\right)C_{T}}\left(\text{activities}\right),\\ C_{crit}\left(pH\right)=\frac{K_{sp}}{\alpha_{2}\left(pH\right)C_{T}}\left(\gamma \equiv 1\right) \end{array}$$

#### 2.1.4 Interpretation

Acidification lowers α_2_(*pH*) and/or reductions in *C*_*T*_ decrease $\left[{CO_{3}}^{2-}\right]=\alpha_{2}C_{T}$, thereby raising *C*_*crit*_(*pH*) (more [*Ca*^2+^] needed to keep Ω = 1).

### 2.2 Mapping of systemic factors

We conducted a targeted literature review to identify systemic and local conditions that influence endolymph [*Ca*^2+^] and *pH*, such as calcium homeostasis disorders, acid–base disturbances, endolymphatic pathological processes, genetic variants, and pharmacological agents (19–33). The mechanistic implications of these factors for CaCO_3_ saturation and otoconia stability are further elaborated in **Section 4.2** of the Discussion.

### 2.3 Simulation study

We conducted an *in silico* simulation to examine how endolymph chemistry modulates otoconia stability. A cohort of *N* = 10,000 virtual individuals was generated. Free calcium values were drawn within 200–350 μM and *pH* within 7.5–7.8, with these windows anchored to vertebrate vestibular reports of [*Ca*^2+^]≈ 250–280 μM and *pH* ≈ 7.6–7.7 (7, 34–37) and extended to encompass plausible pathological deviations related to barrier dysfunction, inflammation, or systemic acid–base disturbance. The total inorganic carbon *C*_*T*_ was calibrated so that *C*_*crit*_ (7.65) = 265 μM, providing a physiologically anchored reference point. Unless otherwise stated, the dispersion parameter in the risk mapping was fixed at σ_*C*_ = 80 μM. To reproduce the empirically right-skewed risk shape with mean *R*≈ 0.68 while keeping the same windows and σ_*C*_, the calcium draw within 200–350 μM was implemented with a mild asymmetry (details and code are provided in Supplementary Data S1); no biological sampling was performed.

For each simulated case, the *pH*-dependent critical calcium concentration *C*_*crit*_(*pH*) was computed from carbonate equilibrium chemistry (pK1 = 6.1, pK2 = 10.3 at ~37 °C; *K*_*sp*_ = 4.47 × 10^−9^). The saturation index was then evaluated as Ω = ($\gamma_{Ca^{2+}}\left[Ca^{2+}\right]\gamma_{{CO_{3}}^{2-}}\left[{CO_{3}}^{2-}\right]$)/*K*_*sp*_; in the minimal model we set activities to unity. Finally, a dimensionless relative risk score was obtained by mapping the calcium distance to threshold through a logistic transform, *R* = 1/{1 + exp (([*Ca*^2+^] – *C*_*crit*_(*pH*))/σ_*C*_)}. By this convention, *R* → 1 indicates undersaturation ([*Ca*^2+^] < *C*_*crit*_, Ω < 1) and a dissolution-prone state, whereas *R* → 0 indicates supersaturation ([*Ca*^2+^] > *C*_*crit*_, Ω>1) and relative mineral stability.

**Figure 1:**
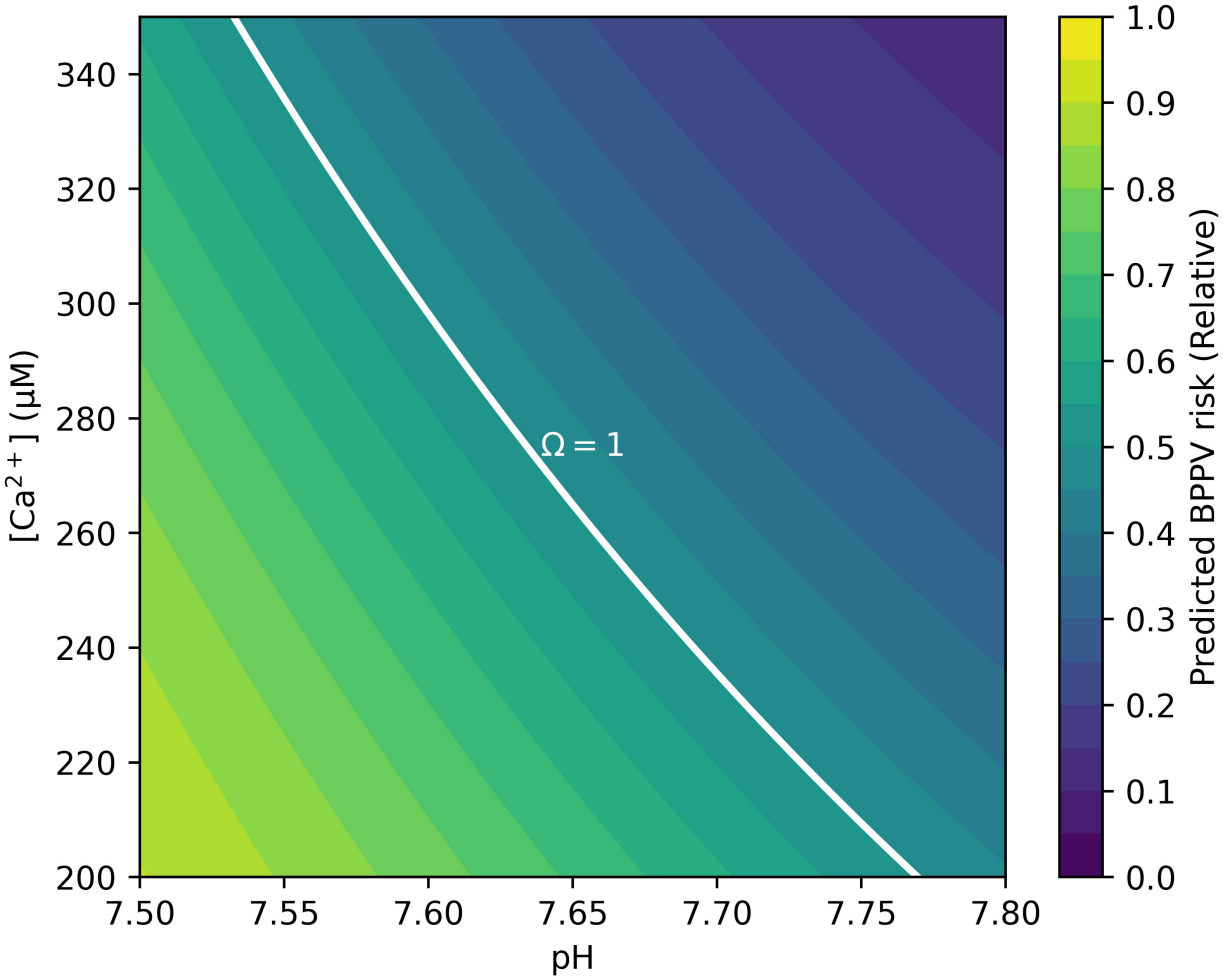
Contour map showing BPPV risk as a function of endolymph *pH* and free calcium [*Ca*^2+^]. The solid white curve denotes the Ω = 1 dynamic-equilibrium boundary, at which precipitation and dissolution of CaCO_3_ are balanced. Regions above this boundary (Ω>1) represent supersaturation and relative otoconia stability, whereas regions below the boundary (Ω < 1) indicate undersaturation and increased dissolution risk. Simulation parameters covered the physiological range of animal vestibular endolymph (250–280 μM [*Ca*^2+^], *pH* 7.6–7.7) and were extended to 200–350 μM and *pH* 7.5–7.8 to encompass plausible pathological deviations. Risk is displayed on the *pH*[*Ca*^2+^] plane; *C*_*T*_, *T*, *I*, and *K*_*sp*_ are held at physiological references; sensitivity analyses are provided in Supplementary Figures.

**Figure 2:**
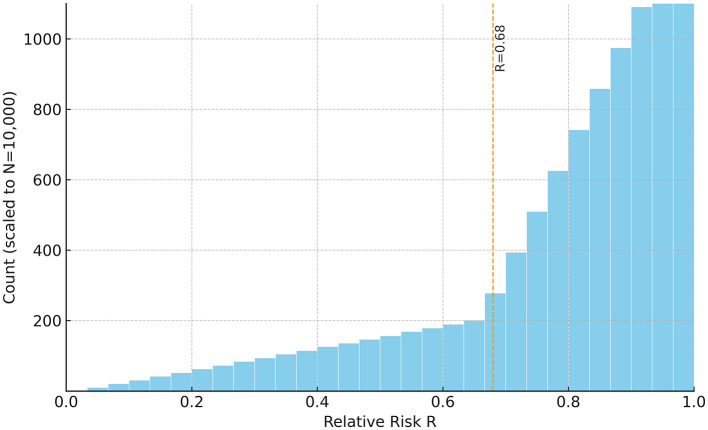
Histogram of simulated relative BPPV risk (*R*) for the synthetic cohort (*N* = 10,000). *R* was computed via Equation 8 with σ_*C*_= 80 μM (i.e., *R* = 1/[1+exp(−Δ*C*/σ_*C*_)], $\text{Δ}C\equiv C_{crit}\left(pH\right)-\left[Ca^{2+}\right]$). Higher *R* ( → 1) indicates undersaturation ([*Ca*^2+^] < *C*_*crit*_; Ω < 1) and greater dissolution propensity, whereas lower *R* ( → 0) reflects supersaturation ([*Ca*^2+^] > *C*_*crit*_; Ω>1) and relative mineral stability. The distribution is right-skewed (mean ≈ 0.78; 80% of cases with *R*> 0.68). The count axis is labeled 0–1,000, and the tallest bar slightly exceeds 1,000. Sampling windows: [*Ca*^2+^] 200–350 μM; *pH* 7.5–7.8; calibration: *C*_*crit*_(7.65)= 265 μM. Interpreting the metrics: a cohort mean of *R*≈0.78 implies that, on average, free calcium lies roughly 100μ*M* (≈10^−4^*M*) below *C*_*crit*_(*pH*); moreover, *R*>0.68 corresponds to ΔC ≳ 60 μM (i.e., $\left[Ca^{2+}\right]-C_{crit}≲-60\mu M$), consistent with a majority occupying the undersaturated (Ω < 1) regime.

Two primary outputs were produced: (i) a risk heatmap on the *pH*[*Ca*^2+^] plane—computed with *C*_*T*_, *T*, *I*, and *K*_*sp*_ held at physiological reference values $\left(T\approx 37^{•}C;\;I=0.20\;M;K_{sp}=4.47\times 10^{-9};C_{T}\;calibrated\;so\;that\;C_{crit}\left(7.65\right)=265\mu M\right)$ with targeted sensitivity analyses—that visualizes Ω and its Ω = 1 boundary separating stable (Ω>1) from unstable (Ω < 1) regions (Figure 1); and (ii) a 30-bin histogram of *R* summarizing the population distribution (Figure 2). The histogram uses a count axis labeled 0–1,000, with the tallest bar slightly exceeding 1,000, making the right-skew explicit. Under the baseline windows and σ_*C*_ = 80 μM, the distribution is right-skewed (mean ≈0.78; 80% with *R*>0.68), consistent with many cases lying just below the saturation boundary near the calibration point (*pH* 7.65, *C*_*crit*_ = 265 μM). The non-linear *pH*-dependence of *C*_*crit*_ and the logistic mapping further accentuate this skew.

Robustness was assessed in a consolidated sensitivity analysis (Supplementary Figure S2). Expanding the windows to [*Ca*^2+^] 180–380 μM or *pH* 7.4–7.9 did not move the Ω = 1 boundary (only the plotted axes were extended). Varying σ_*C*_ between 40 and 120 μM steepened or flattened the logistic mapping of *R* without shifting Ω = 1, consistent with the boundary's independence from the logistic transform. In contrast, scaling *C*_*T*_ by ±10% displaced the Ω = 1 boundary modestly while preserving the qualitative risk-contour structure. For completeness, ionic-strength effects computed with Davies activity coefficients at ~37 °C are shown in Supplementary Figure S1: increasing *I* (0.15, 0.20, 0.30 M) lowers γ and shifts the Ω = 1 boundary upward (higher [*Ca*^2+^] required to maintain Ω = 1) across *pH* 7.4–7.9, while the qualitative topology is preserved.

#### 2.3.1 Reproducibility

All analyses were performed *in silico*; the synthetic cohort (*N* = 10,000) and plotting code are provided as Supplementary Data S2 and reproduce Figure 2 under the parameters described herein (*C*_*T*_ calibrated so that *C*_*crit*_ (7.65) = 265 μM; σ_*C*_ = 80 μM; *pH* 7.5–7.8; [*Ca*^2+^] 200–350 μM).

### 2.4 Core mathematical method

We formulate a deterministic, three-step mapping from (*pH*, [*Ca*^2+^]) to (Ω, *R*); no empirical training is required.

Step 1—Carbonate speciation. Given *pH*, carbonate dissociation constants (*pK*_1_, *pK*_2_) define the fractional species α_0_(*pH*), α_1_(*pH*), α_2_(*pH*). The carbonate available for precipitation is


$$\begin{array}{l} \left[CO_{3}^{2-}\right]=\alpha_{2}\left(pH\right)C_{T} \end{array}$$

where *C*_*T*_ is total inorganic carbon (see **Section 2.1** for definitions and constants).

Step 2—Saturation index. The carbonate saturation index is


$$\begin{array}{l} \text{Ω}\approx \frac{\left[Ca^{2+}\right]\left[{CO_{3}}^{2-}\right]}{K_{sp}},\;\gamma \equiv 1 \end{array}$$

Step 3—Logistic risk mapping.

Let


$$\begin{array}{l} \text{Δ}C\equiv C_{crit}\left(pH\right)-\left[Ca^{2+}\right],C_{crit}\left(pH\right)=\frac{K_{sp}}{\alpha_{2}\left(pH\right)C_{T}} \end{array}$$

The probability-like, dimensionless risk score is


$$\begin{array}{l} R=\frac{1}{1+\exp\left(-\text{Δ}C/\sigma_{C}\right)} \end{array}$$

so that *R* = 0.5 at Δ*C* = 0 (i.e., Ω = 1), *R* → 1 for Δ*C*>0 (undersaturation), and *R* → 0 for Δ*C* < 0 (supersaturation).

#### 2.4.1 Calibration and separation of roles

For simulations we scale *C*_*T*_ so that *C*_*crit*_(*pH* = 7.65) = 265μ*M* (physiological anchor). The CO_2_ solubility coefficient is not used in theoretical simulations; it is invoked only in the clinical translation pipeline when inferring *C*_*T*_ from blood-gas variables. This separation keeps the theoretical equations transparent while clarifying how clinical inputs are mapped onto the same framework.

#### 2.4.2 Clinical translation using a blood-derived proxy

To validate directional and temporal predictions without endolymph sampling, we define a blood-derived Ω proxy from serum ionized calcium and blood-gas $pH/HCO_{3}^{-}/pCO_{2}$. The proxy is intended to test associations (e.g., BPPV status) and within-person phase concordance, not to estimate absolute endolymph states. All claims of clinical applicability are therefore conditional on prospective calibration of blood– endolymph relationships.

## 3 Results

The model predicted that systemic factors influencing *pH* or [*Ca*^2+^] produce distinct shifts in the carbonate saturation index (Ω) and thereby alter otoconia stability. Increases in *pH*–as in hyperventilation, prolonged vomiting, or with thiazide diuretics—elevated Ω. Increases in [*Ca*^2+^]–e.g., in hyperparathyroidism or vitamin D excess—also raised Ω. Conversely, decreases in *pH*–as in metabolic or respiratory acidosis, and with acetazolamide—lowered Ω. Loop diuretics reduced [*Ca*^2+^] and, when combined with volume depletion, further depressed Ω. Likewise, hypocalcemic states (e.g., vitamin D deficiency or hypoparathyroidism) reduced Ω.

Simulation outputs were consistent with these predictions. The risk map showed that low *pH* combined with low [*Ca*^2+^] markedly expanded the Ω < 1 region (Figure 1). The Ω = 1 contour cleanly delineated the transition between stable (Ω>1)and unstable (Ω < 1) states. The histogram of relative risk scores (*R*) was right-skewed with mean ≈0.78, and 80% of the cohort had *R*>0.68, indicating that a large fraction of the synthetic population resides near or below the saturation boundary (Figure 2). Consistent with the plotting parameters of Figure 2, the count axis was labeled 0–1,000 and the tallest bar slightly exceeded 1,000, making the right-skew visually explicit.

To aid clinical interpretation, we also examined a blood-derived proxy: hypocalcemia (e.g., ionized calcium ≲ 0.96 mM, with severe cases ~0.68 mM) or acidemia (*pH* 7.30–7.35) typically corresponds to Ω < 1 under the minimal model assumptions and therefore maps to high *R*. This proxy is illustrative rather than a direct measurement of endolymph chemistry, and we emphasize that definitive inferences require vestibular- compartment data.

Further shifts of the Ω = 1 boundary under *K*_*sp*_ perturbations (±10–20%) and temperature (35–39 °C; with *pK*_1_(*T*), *pK*_2_(*T*), and *K*_*sp*_(*T*) updated) are provided in Supplementary Figures S3, S4. These analyses preserved the qualitative topology of the boundary while moving its position modestly. A consolidated sensitivity analysis (Supplementary Figure S2) showed that broadening the sampling windows to [*Ca*^2+^] 180–380 μM or *pH* 7.4–7.9 did not move Ω = 1 (only the plotted axes enlarged). Varying σ_*C*_ between 40 and 120 μM steepened or flattened the logistic mapping from threshold distance to *R* without shifting Ω = 1, consistent with the boundary's independence from the risk transform.

Reproducibility. All analyses were performed *in silico*; the synthetic cohort (*N* = 10, 000) and plotting code are provided as Supplementary Data S2 and exactly regenerate Figure 2 using the baseline parameter set described in **Section 2.3**.

## 4 Discussion

Human otoconia are calcite-based CaCO_3_ biominerals, so the carbonate saturation-index framework used in crystallization and biomineralization applies naturally to the vestibular system. We therefore adopted the physically interpretable saturation index Ω (Equation 1) as the core descriptor of the precipitation–dissolution balance and evaluated it under endolymphatic conditions (16, 17). Although many marine skeletons (e.g., corals, mollusks) precipitate aragonite rather than calcite, the Ω formalism remains applicable; differences arise through *K*_*sp*_ and kinetic pathways. In this study, *K*_*sp*_ was treated as an effective constant at ~37 °C and physiological ionic strength, and—in the minimal model—activities were approximated by concentrations. Potential departures from ideality (e.g., organic-matrix mediation, non-ideal solution effects) are addressed in **Section 4.6** (Limitations and Future Directions).

**Figure 3:**
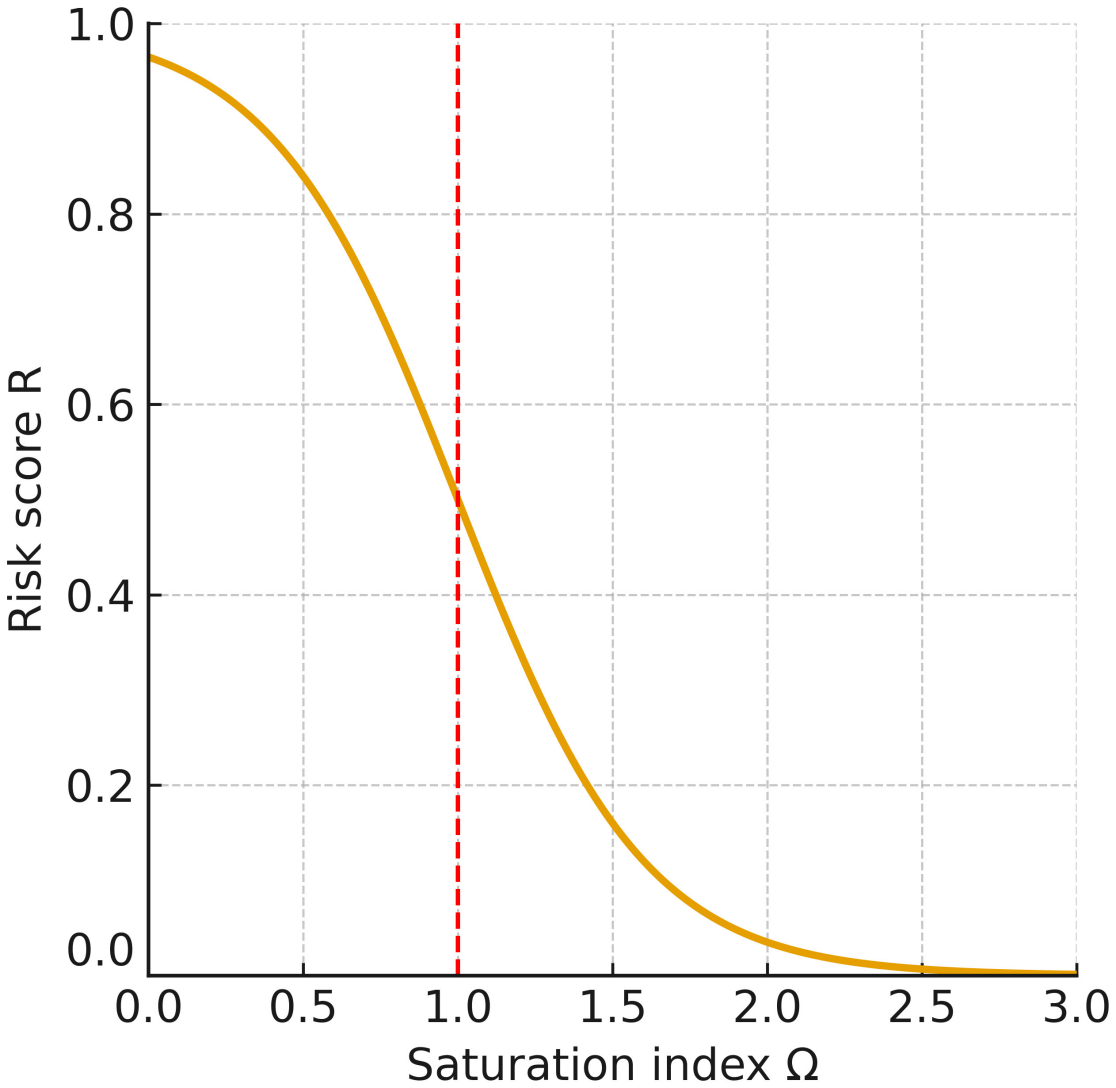
Relationship between the saturation index (Ω) and risk score (*R*). At the equilibrium point (Ω = 1), the risk is *R* = 0.5. When Ω falls below 1, *R* rises steeply toward 1, indicating a dissolution-prone state for otoconia and a higher likelihood of recurrent or persistent BPPV; when Ω exceeds 1, *R* declines toward 0, reflecting a stable condition in which otoconia are preserved. This monotonic relationship shows that even a modest reduction of Ω below 1 sharply increases the predicted risk.

Building on this formulation, we constructed a minimal ion–chemistry model that integrates endolymphatic [*Ca*^2+^] and *pH* into a quantitative assessment of BPPV risk. Expressing otoconia stability through Ωcaptures the dynamic balance between CaCO_3_ precipitation and dissolution, the principal mineral process governing otoconia. Because carbonate speciation links Ω non-linearly to [*Ca*^2+^] and *pH*, even modest perturbations can drive the system toward net dissolution (Ω < 1) or net precipitation (Ω>1; Figure 3). Although endolymph is generally maintained in a supersaturated state (Ω>1), its buffering capacity is lower than that of cerebrospinal fluid, rendering it more vulnerable to systemic or local disturbances in acid–base balance or calcium homeostasis (38). Consistent with this susceptibility, our simulations produced a right-skewed distribution of risk scores *R* (Figure 2) and clear, interpretable Ω = 1 boundaries in the *pH*[*Ca*^2+^] plane (Figure 1), linking biochemical shifts directly to predicted mechanical stability of otoconia.

### 4.1 Mechanistic implications

At Ω = 1, precipitation and dissolution of CaCO_3_ are in dynamic equilibrium, so otoconial mass is preserved on average while ion exchange continues. The system is highly sensitive: small changes in *pH* or [*Ca*^2+^] can push it toward net precipitation (Ω>1) or net dissolution (Ω < 1), with disproportionate effects on otoconia integrity and detachment risk (Figure 3). The equilibrium calcium threshold *C*_*crit*_(*pH*) defined in Equation 10 makes this dependence explicit: modest acidification (which lowers α_2_) or reduced calcium availability readily depresses Ω below unity, predisposing to instability. In our risk mapping (Equation 8), this appears as $\text{Δ}C=C_{crit}\left(pH\right)-\left[Ca^{2+}\right]$: Δ*C*>0 yields *R* near 1 (undersaturation), whereas Δ*C* < 0 yields *R* near 0 (supersaturation).

The inherent chemical vulnerability of otoconia arises from the weaker ionic bonding of calcite CaCO_3_ compared with hydroxyapatite *Ca*_10_(*P*_*O*_4_)6_(*OH*)_2_. Hydroxyapatite has a highly stable lattice and very low solubility product, rendering it resistant to physiological *pH*fluctuations. By contrast, CaCO_3_ dissolves more readily as *pH* decreases because carbonate equilibria shift toward $HCO_{3}^{-}$ and dissolved *CO*_2_. Utricular endolymph is typically ~ *pH* 7.6–7.7 (7, 34–37), so even mild acidotic shifts can depress Ω below unity and raise CaCO_3_ solubility.

### 4.2 Convergence of etiologies and clinical alignment

A wide range of systemic and local conditions ultimately converge on a single chemical pathway that governs otoconia stability: modulation of the carbonate saturation index, Ω. Disturbances of calcium homeostasis—whether from hyper- or hypocalcemia, vitamin D deficiency or excess, or hyper-/hypoparathyroidism—shift free endolymphatic [*Ca*^2+^] and thereby alter Ω (19–22). Acid–base disorders operate through carbonate speciation α_2_(*pH*): metabolic or respiratory acidosis lowers *pH*, reduces α_2_, and depresses Ω, whereas alkalosis has the opposite effect (23, 24). Disease processes such as vestibular migraine, Ménière's disease, autoimmune inner-ear disease, and viral inner-ear disorders can change endolymph composition and destabilize otoconia (25, 26). Genetic factors encompass channelopathies and transporter defects affecting endolymph homeostasis and migraine susceptibility. Variants in *CACNA1A* (P/Q-type voltage-gated Ca^2+^ channel α1A subunit) and *ATP1A2* (Na^+^/K^+^-ATPase α2 subunit) primarily alter Ca^2+^ handling in vestibular epithelia, leading to changes in free endolymphatic [*Ca*^2+^] and otoconia stability. Mutations in *SLC26A4* (pendrin, ${\text{Cl}}^{+}/HCO_{3}^{-}$ exchanger) and *SLC4A11* (H^+^/OH^−^ transporter) predominantly affect pH and bicarbonate balance, shifting carbonate equilibrium (α_2_) and thereby indirectly influencing CaCO_3_ saturation (Ω) (27–31). Pharmacological agents also steer Ω in predictable directions: thiazides tend toward metabolic alkalosis and raise Ω; acetazolamide induces metabolic acidosis and lowers Ω; loop diuretics reduce [*Ca*^2+^] and, with volume depletion, further depress Ω (32, 33).

Viewed through Equation 1, these influences either decrease the activity product $\gamma_{Ca^{2+}}\left[Ca^{2+}\right]\gamma_{{CO_{3}}^{2-}}\left[{CO_{3}}^{2-}\right]$ (for example by lowering [*Ca*^2+^] or α_2_(*pH*), or by increasing ionic strength and thereby lowering activity coefficients during labyrinthitis, autoimmunity, or barrier breakdown), or else increase it and move the system away from the dissolution threshold (22, 39–47). Repetitive neurogenic inflammation in vestibular migraine or Ménière's disease may additionally disrupt *Ca*^2+^ transporters in dark cells, transiently elevating free [*Ca*^2+^]; concurrent degradation of otoconial matrix proteins then impairs lattice incorporation and weakens stability (26, 48). Combinations of insults are particularly consequential: acidosis coupled with labyrinthitis can drive Ω well below 1 and accelerate CaCO_3_ dissolution, whereas alkalosis with elevated [*Ca*^2+^] can push Ω well above 1, stabilizing otoconia but potentially favoring pathological crystal aggregation. Taken together, these convergent mechanisms destabilize the protein–mineral composite of otoconia and yield fragments more prone to detachment during head movements, aligning the chemical predictions with clinical vulnerability patterns (Figure 1).

### 4.3 Alignment with clinical and epidemiological data

The model's predictions are consistent with a broad spectrum of clinical and epidemiological findings. Higher recurrence rates in post-menopausal women and in patients with osteoporosis reflect hormonally mediated reductions in calcium homeostasis, which also explain the greater prevalence observed in older adults and in women. Seasonal variation in BPPV incidence, particularly during winter months when vitamin D deficiency is more prevalent, further supports the predicted vulnerability of otoconia under conditions of impaired calcium absorption (49).

Using the blood-derived proxy for Ω, the model predicts that when laboratory surrogates fall outside customary reference ranges (e.g., *pH* ~7.30–7.35 or ionized calcium ~0.68–0.96 mM), cases often map to the dissolution-prone domain (Ω ≤ 1) with high risk (frequently *R*≥0.8). In our baseline mapping, 80% of the cohort had *R*>0.68. These thresholds emphasize that even modest systemic deviations can destabilize otoconia and are consistent with reports of persistent or recurrent dizziness in patients with metabolic or acid–base disturbances.

For comparative interpretation at fixed *K*_*sp*_, we introduced a dimensionless relative critical-calcium index (Equation 9), *I*_*C*_*crit*__∝1/(α_2_(*pH*)*C*_*T*_). Within the simulated endolymph *pH* range (7.5–7.8), the index is on the order of 0.06–0.20: it rises toward ~0.2 with acidosis (lower α_2_) and falls toward ~0.06 with alkalosis, providing a simple surrogate link between systemic acid–base status and inner-ear carbonate equilibrium. Importantly, this framework offers a clinically feasible bridge from standard blood-gas analysis to risk stratification without invasive endolymph sampling. Where relevant, changes in ionic strength should be interpreted as activity effects (via γ) that modify the activity product in Equation 1 rather than *K*_*sp*_ itself (see Supplementary Figure S1 for Davies-based illustrations).

Beyond chronic factors, the onset of BPPV after systemic illness or prolonged bed rest can be interpreted—among other mechanisms—as a plausible consequence of respiratory acidosis. Reduced ventilatory drive and CO_2_ retention during inactivity may lower blood *pH*, depress α_2_, and thereby lower Ω, promoting CaCO_3_ dissolution (50, 51). Immobilization-related changes in bone turnover may further perturb systemic calcium balance. Frequent recurrence after apparent resolution is likewise compatible with the notion that many patients reside near the Ω = 1 equilibrium boundary, where modest biochemical perturbations can tip the balance toward instability. Reported ranges of [*Ca*^2+^] and *pH* across these conditions align with simulation-derived zones of instability, underscoring the translational relevance of the saturation-index framework in linking mechanistic predictions to observed clinical patterns.

### 4.4 Clinical translation and preventive potential

Because the model is deterministic and requires only two inputs ([*Ca*^2+^], *pH*), it can be operationalized with serum ionized calcium and blood gases as non-invasive surrogates. Crucially, these blood-based variables are not one-to-one estimators of vestibular endolymph; they support relative-risk inference via a blood-derived Ω proxy, reflecting directional trends rather than absolute endolymph states. This stance follows from the practical infeasibility of direct human utricular/saccular sampling and is especially useful for individualized risk stratification in patients with recurrent BPPV or elevated baseline risk (e.g., osteoporosis, chronic kidney disease, migraine, Ménière's disease).

To facilitate translation while respecting this limitation, we pre-specify two indirect validation designs. (i) Cross-sectional case–control: test whether a blood-derived Ω proxy (from ionized calcium and blood-gas–derived $pH/HCO_{3}^{-}/pCO_{2}$, temperature-corrected to 37 °C; arterial preferentially, venous acceptable with caveats) is independently associated with prevalent BPPV after adjustment for confounders (age, sex, BMI, renal function, vitamin D/PTH, diabetes/respiratory disease, diuretic/acetazolamide/PPI use, and hydration). (ii) Repeated-measures cohort: evaluate temporal concordance between within-person fluctuations in the proxy and symptom phases (exacerbation, resolution, and post-repositioning residual dizziness) using mixed-effects models and lagged windows (e.g., ±24–48 h). In both designs, inference is limited to association and timing, not absolute endolymph chemistry.

We also commit to robustness checks around key physicochemical assumptions: activity-coefficient/ionic-strength variation (e.g., extended Debye–Hückel within Pitzer-bounded ranges), *K*_*sp*_ within physiologic intervals, *C*_*T*_ estimated from $HCO_{3}^{-}/pCO_{2}$ rather than fixed, and temperature within clinical limits. These analyses quantify boundary shifts around Ω = 1 and test the stability of relative-risk mapping under reasonable biochemical uncertainty.

The framework provides a mechanistic lens on post-repositioning dizziness and the clinical heterogeneity of BPPV. After apparently successful canalith repositioning, endolymph may transiently reside near the metastable Ω≈1 regime, in which precipitation and dissolution are finely balanced; otoconia are neither fully stabilized nor fully dissolved, yielding residual vestibular instability perceived clinically as lingering disequilibrium rather than true vertigo (52). More broadly, BPPV spans a continuum of otoconial states, from intact crystalline otoliths to partially dissolved or fragmented particles, which helps explain absent nystagmus, atypical directions/durations of positional responses, and subtle imbalance without overt vertigo (53–55).

Bone homeostasis reflects slow hydroxyapatite turnover over months to years; by contrast, human otoconia are calcite-based, and their stability depends on the carbonate saturation index $\text{Ω}(\approx [Ca^{2+}][CO]/K_{sp}$ in the minimal model). Because α_2_(*pH*) and [*Ca*^2+^] modulate Ω on short timescales, transient shifts in ionized calcium and acid–base status can promptly tilt otoconia toward dissolution. Thus, while low BMD and hypocalcemic states index chronic susceptibility, ionized calcium is expected to track near-term BPPV vulnerability more closely than BMD or total calcium (22, 56)—a hypothesis pending the prospective validation outlined above.

Preventive and therapeutic implications suggested by the model include maintaining acid–base neutrality during acute illness or the post-operative period, correcting vitamin D or calcium deficiency while avoiding excessive supersaturation, and judicious pharmacologic modulation of vestibular ion transport in channelopathy settings—e.g., thiazides (alkalosis → Ω↑) or acetazolamide (acidosis → Ω↓) vs. loop diuretics ([*Ca*^2+^]↓ → Ω↓) (57–60). A practical advantage is that the Ω proxy is computable entirely from clinically accessible data, obviating hazardous endolymph sampling, while the pre-specified validation and robustness testing provide the necessary bridge toward eventual clinical use.

### 4.5 Pharmacological modulation and hydration balance

Clinically, excessive dehydration can raise ionic strength and thereby alter ion activities (via the activity coefficients, γ), which lowers the activity product in Equation 1 and—particularly under acidic conditions—can accelerate CaCO_3_ dissolution.

Within this framework, drug classes have predictable directions of effect. Thiazide diuretics tend to promote calcium retention and metabolic alkalosis, thereby increasing α_2_(*pH*) and raising Ω (57, 58). By contrast, acetazolamide (carbonic-anhydrase inhibition) typically induces metabolic acidosis, lowering α_2_(*pH*) and thus decreasing Ω–useful for fluid dynamics in Ménière's but destabilizing for carbonate chemistry in our model (59). Loop diuretics increase calciuresis and, when coupled with dehydration, reduce free [*Ca*^2+^] and can depress Ω (58, 60); however, their contraction alkalosis tends to increase α_2_(and Ω), so the net effect depends on fluid and electrolyte management.

Clinical application aims to maintain eubicarbonatemia and normocalcemia so that endolymph remains mildly supersaturated (Ω>1). Preferential use of alkalinizing or calcium-sparing strategies (e.g., thiazides where appropriate), adequate hydration, and correction of vitamin D and calcium deficits can mitigate dissolution risk. If loop diuretics are unavoidable, they should be paired with careful fluid/electrolyte monitoring (including ionized calcium and acid–base status). Framed this way, endolymphatic pressure control aligns with preservation of the carbonate equilibrium that supports otoconia integrity.

### 4.6 Limitations and future directions

This minimal model reduces inner-ear chemistry to a saturation index Ω computed from ionized calcium [*Ca*^2+^] and *pH* and maps it to a probability-like risk score via a logistic transform. Its usability relies on deliberate simplifications: *K*_*sp*_ is treated as an effective constant at 37 °C and physiological ionic strength; activities are approximated by concentrations; calcite is assumed as the operative polymorph (polymorph-dependent kinetics not modeled); baseline total inorganic carbon *C*_*T*_ is held fixed; and spatial heterogeneity, time-dependent transport, and chemo–mechanical coupling (detachment forces, otolithic-membrane pathology, and aggregation) are omitted. Organic-matrix effects and explicit protein–ion interactions—known to influence nucleation, stabilization, and dissolution—are likewise not yet incorporated. These choices were intentional to provide a transparent, reproducible, and falsifiable starting point.

A central limitation is the practical infeasibility of direct human utricular/saccular endolymph sampling, precluding empirical measurement of absolute vestibular chemistry. Consequently, our framework is hypothesis-generating, and all clinical inferences are restricted to what can be supported by blood-based surrogates. Specifically, we construct a blood-derived Ω proxy—from serum ionized calcium and blood-gas–derived $pH/HCO_{3}^{-}$/pCO_2_–to test directionality (association with BPPV) and temporal concordance (within-person fluctuation vs. symptom phases), not to estimate absolute endolymph states. In the absence of direct human data, our parameter ranges were therefore anchored to vertebrate vestibular studies, which consistently report utricular [*Ca*^2+^] around 250–280 μM and *pH* ~7.6–7.7 (34–37). By contrast, Bächinger et al. (61) reported endolymph calcium concentrations of 0.017–0.133 mmol·L^−1^ (17–133 μM), values largely derived from animal endolymphatic sac and cochlear measurements, and inferred that similar mechanisms may operate in humans (61). These values cannot be directly compared with vestibular (utricle/saccule) endolymph concentrations, underscoring the importance of clearly specifying the anatomical compartment when modeling BPPV.

#### 4.6.1 Sensitivity analyses

We implemented four sensitivity sweeps—(i) activity/ionic-strength assumptions (Davies activity coefficients with Pitzer-bounded ranges; note that ionic strength changes Ω by altering ion activities via γ, i.e., the activity product in Equation 1, rather than *K*_*sp*_ itself); (ii) total inorganic carbon *C*_*T*_ (±10%); (iii) the solubility product *K*_*sp*_ (±10%−20%); and (iv) temperature (35–39 °C), updating *pK*_1_(*T*), *pK*_2_(*T*), *K*_*sp*_(*T*), and CO_2_ solubility accordingly. Across all four, the Ω = 1 boundary shifted only modestly, and the qualitative dissolution-prone topology was preserved. Boundary displacements were most pronounced under activity/ionic-strength (Supplementary Figure S1) and *K*_*sp*_ variations (Supplementary Figure S3), intermediate for *C*_*T*_ (Supplementary Figure S2), and smallest for temperature within the clinical range examined (Supplementary Figure S4). As expected, the Ω = 1 boundary is independent of logistic-mapping parameters (e.g., dispersion σ_*C*_). These findings support the qualitative robustness of the framework while highlighting where quantitative calibration will matter most for clinical translation.

Future work will: (i) implement activity-aware Ω using bounded Pitzer ranges for ion–ion interactions (62, 63); (ii) estimate *C*_*T*_ from measured bicarbonate and *pCO*_2_ rather than assuming it fixed; (iii) add carbonate–protein interactions and matrix effects to refine nucleation/dissolution kinetics; and (iv) couple the chemical module to biomechanical simulations of otoconia dynamics to build a multiphysics description of BPPV. Ultimately, prospective, surrogate-based calibration in humans (using serum ionized calcium and blood-gas–derived $pH/HCO_{3}^{-}$/pCO_2_, together with clinical outcomes) will be required before any clinical deployment.

Although the framework omits organic-matrix effects, explicit protein–ion interactions, and mechanical forces, its qualitative predictions are expected to hold for human otoconia: because human otoconia are composed of calcium carbonate, the sharp rise in dissolution risk once Ω < 1 is a basic physicochemical property, not a model artifact (see Figure 3). Accordingly, specific numerical values (e.g., *C*_*crit*_) and contour shapes should be viewed as illustrative, whereas near-boundary behavior around Ω≈1 is likely to be valid. The model should therefore be regarded as hypothesis-generating, with prospective calibration and incorporation of additional mechanisms required for quantitative validation. Our sensitivity analyses further support this view, showing only modest shifts of the Ω = 1 boundary under physiologically plausible parameter variations while preserving the qualitative dissolution-prone topology.

## 5 Conclusion

Canalith repositioning maneuvers remain the cornerstone of care for benign paroxysmal positional vertigo (BPPV). Yet the calcium-carbonate composition of human otoconia—unlike the phosphate-based mineral of bone—renders them chemically fragile and sensitive to relatively small ionic and *pH* perturbations (43). Beyond the established mechanical framework of BPPV— comprising the canalithiasis and cupulolithiasis mechanisms—we introduce an Ω-based biochemical model (*pH*, [*Ca*^2+^]) that delineates the otoconia stability–dissolution boundary (Ω≈1) and complements—rather than replaces—this framework.

A minimal, deterministic ion–chemistry framework formalizes this fragility through the carbonate saturation index Ω, which integrates ionized calcium and *pH*-dependent carbonate speciation. When Ω falls below unity, dissolution predominates, predisposing otoconia to fragmentation and detachment. Given its deliberate parsimony and reliance on synthetic data, the framework is intended as a hypothesis-generating construct rather than a validated clinical tool.

This perspective links systemic influences—such as vitamin D deficiency, estrogen decline, acidosis, and endolymphatic inflammation—to otoconia vulnerability and recurrent BPPV risk. In this translational pathway, serum ionized calcium together with blood-gas–derived $pH/HCO_{3}^{-}$/pCO_2_ are positioned only as non-invasive surrogates for relative-risk inference (a blood-based Ω proxy), rather than one-to-one estimators of absolute endolymph chemistry. Prospective, surrogate-based validation and calibration in humans—along with robustness testing to variations in *C*_*T*_, ionic strength/activity coefficients, *K*_*sp*_, and temperature within physiological bounds—are required before any clinical deployment.

Finally, coupling this chemical module to biomechanical simulations of otoconia dynamics offers a route toward a multiphysics description of BPPV pathophysiology. A prospectively validated, activity-aware Ω (with bounded Pitzer ranges) integrated into such a framework may ultimately provide a quantitative bridge between vestibular biochemistry and clinical management; at present, the model is best viewed as a clear, testable basis for future studies.

## Data Availability

The datasets generated for this study are not publicly available because they consist of synthetic simulation outputs rather than primary experimental data. They are available from the corresponding author upon reasonable request.

## Supplementary material

Supplementary Figure S1 Effect of ionic strength on the Ω = 1 boundary in the [*Ca*^2+^]- *pH* plane using Davies activity coefficients γ at ~37 °C. Curves show Ω = 1 for *I* = 0.15, 0.20, 0.30 M; the calibration point ( × ) is set to Ω = 1 at *pH* = 7.65, [*Ca*^2+^] = 265 μ*M*, *I* = 0.20 M by adjusting *C*_*T*_. Increasing ionic strength lowers γ and shifts the boundary upward (higher [*Ca*^2+^] required to maintain Ω = 1) across *pH* 7.4–7.9, while the qualitative topology is preserved.

Supplementary Figure S2 Consolidated sensitivity analysis. Baseline risk map *R* in the *pH*[*Ca*^2+^] plane with Ω = 1 boundaries overlaid for *C*_*T*_ scaling of −10% (blue dashed) and +10% (red dashed), in addition to the baseline (black solid). The Ω = 1 boundary shifts modestly with *C*_*T*_ variation, while the qualitative risk-contour structure is preserved. Changes in σ_*C*_ (40–120 μM), expansion of the [*Ca*^2+^] range (200–350 → 180–380 μM), and extension of the *pH* range (7.5–7.8 → 7.4–7.9) did not alter the number of Ω = 1 boundaries, affecting only axis scaling or the steepness of the logistic mapping (see Methods 2.3). For comparison, plausible blood–endolymph offsets (blood [*Ca*^2+^] ~1.0–1.3 mM vs. utricle/saccule endolymph [*Ca*^2+^] 250–280 μM, *pH* ~7.6–7.7) would quantitatively shift the Ω = 1 boundary, but the qualitative dissolution-prone pattern remains unchanged.

Supplementary Figure S3 Ω = 1 boundary as a function of *pH* under *K*_*sp*_ variations (±10–20%) at ~37 °C and *I* = 0.20 M. Curves show *K*_*sp*_ **×** {0.80, 0.90, 1.00, 1.10, 1.20}; the calibration point ( × ) denotes Ω = 1 at *pH* 7.65 and [*Ca*^2+^] = 265μ*M*. Increasing *K*_*s*_ shifts the boundary upward (higher [*Ca*^2+^] required to maintain Ω = 1), while the qualitative topology is preserved.

Supplementary Figure S4 Ω = 1 boundary vs. *pH* at 35–39 °C, updating *pK*_1_(*T*), *pK*_2_(*T*) and *K*_*sp*_(*T*) (illustrative linearized adjustments) with *I* = 0.20 M. The calibration point (×) is set at 37 °C. Within this clinical range, higher temperature slightly lowers the required [*Ca*^2+^] to maintain Ω = 1, with the qualitative structure retained.
