# Supplementary figures and images for: A minimal ion–chemistry model for predicting benign paroxysmal positional vertigo risk based on endolymphatic calcium and pH
Source: Front Neurol. 2025 Oct 23;16:1690931. doi: 10.3389/fneur.2025.1690931 (PMC12590773; doi:10.3389/fneur.2025.1690931)

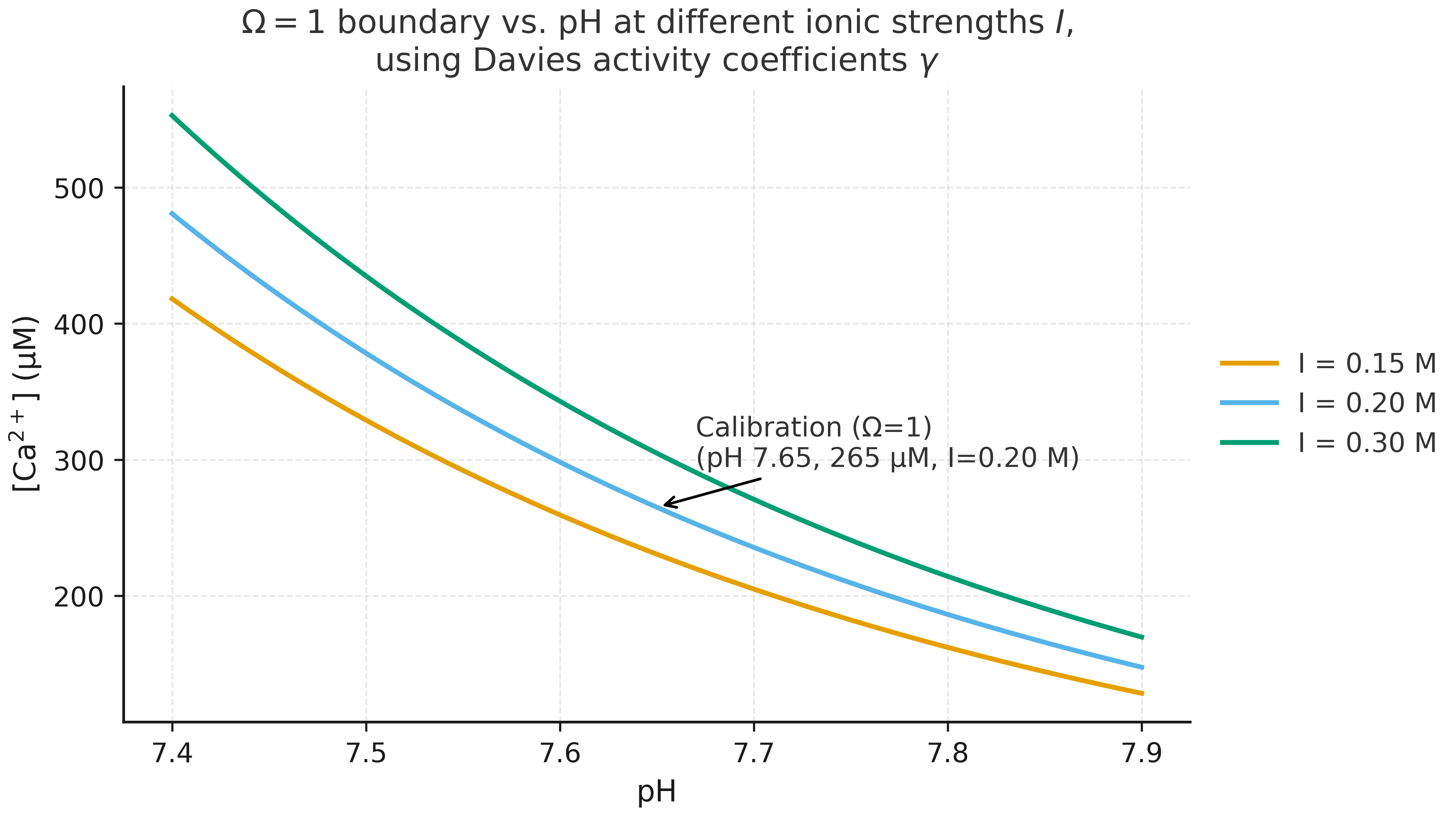

Supplement: Supplementary Figure S1 — Effect of ionic strength on the Ω = 1 boundary in the [Ca2+]- pH plane using Davies activity coefficients γ at ~37 °C. Curves show Ω = 1 for I = 0.15, 0.20, 0.30 M; the calibration point ( × ) is set to Ω = 1 at pH = 7.65, [Ca2+] = 265 μM, I = 0.20 M by adjusting CT. Increasing ionic strength lowers γ and shifts the boundary upward (higher [Ca2+] required to maintain Ω = 1) across pH 7.4–7.9, while the qualitative topology is preserved. [file Image_1.tif]

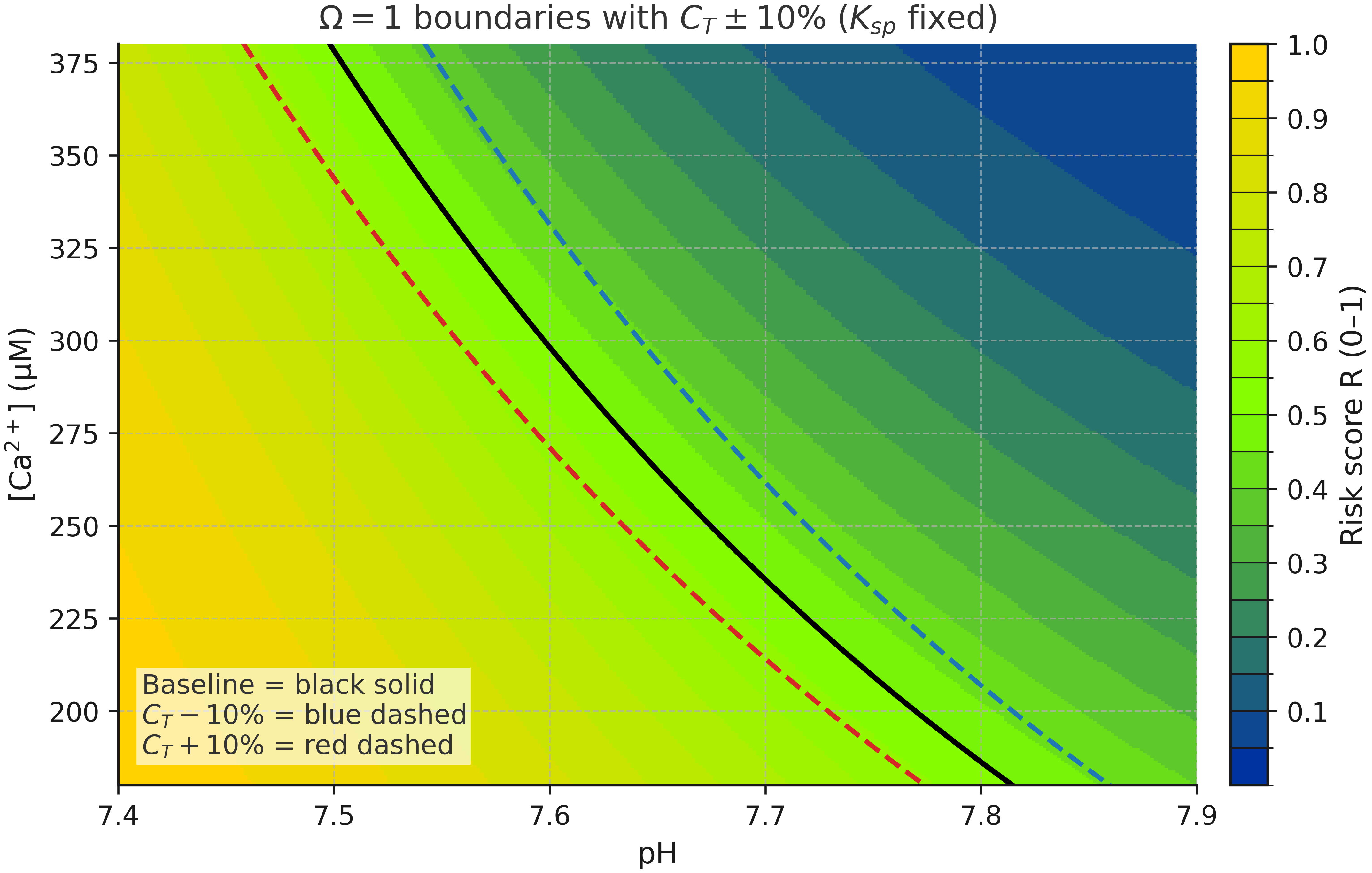

Supplement: Supplementary Figure S2 — Consolidated sensitivity analysis. Baseline risk map R in the pH[Ca2+] plane with Ω = 1 boundaries overlaid for CT scaling of −10% (blue dashed) and +10% (red dashed), in addition to the baseline (black solid). The Ω = 1 boundary shifts modestly with CT variation, while the qualitative risk-contour structure is preserved. Changes in σC (40–120 μM), expansion of the [Ca2+] range (200–350 → 180–380 μM), and extension of the pH range (7.5–7.8 → 7.4–7.9) did not alter the number of Ω = 1 boundaries, affecting only axis scaling or the steepness of the logistic mapping (see Methods 2.3). For comparison, plausible blood–endolymph offsets (blood [Ca2+] ~1.0–1.3 mM vs. utricle/saccule endolymph [Ca2+] 250–280 μM, pH ~7.6–7.7) would quantitatively shift the Ω = 1 boundary, but the qualitative dissolution-prone pattern remains unchanged. [file Image_2.tif]

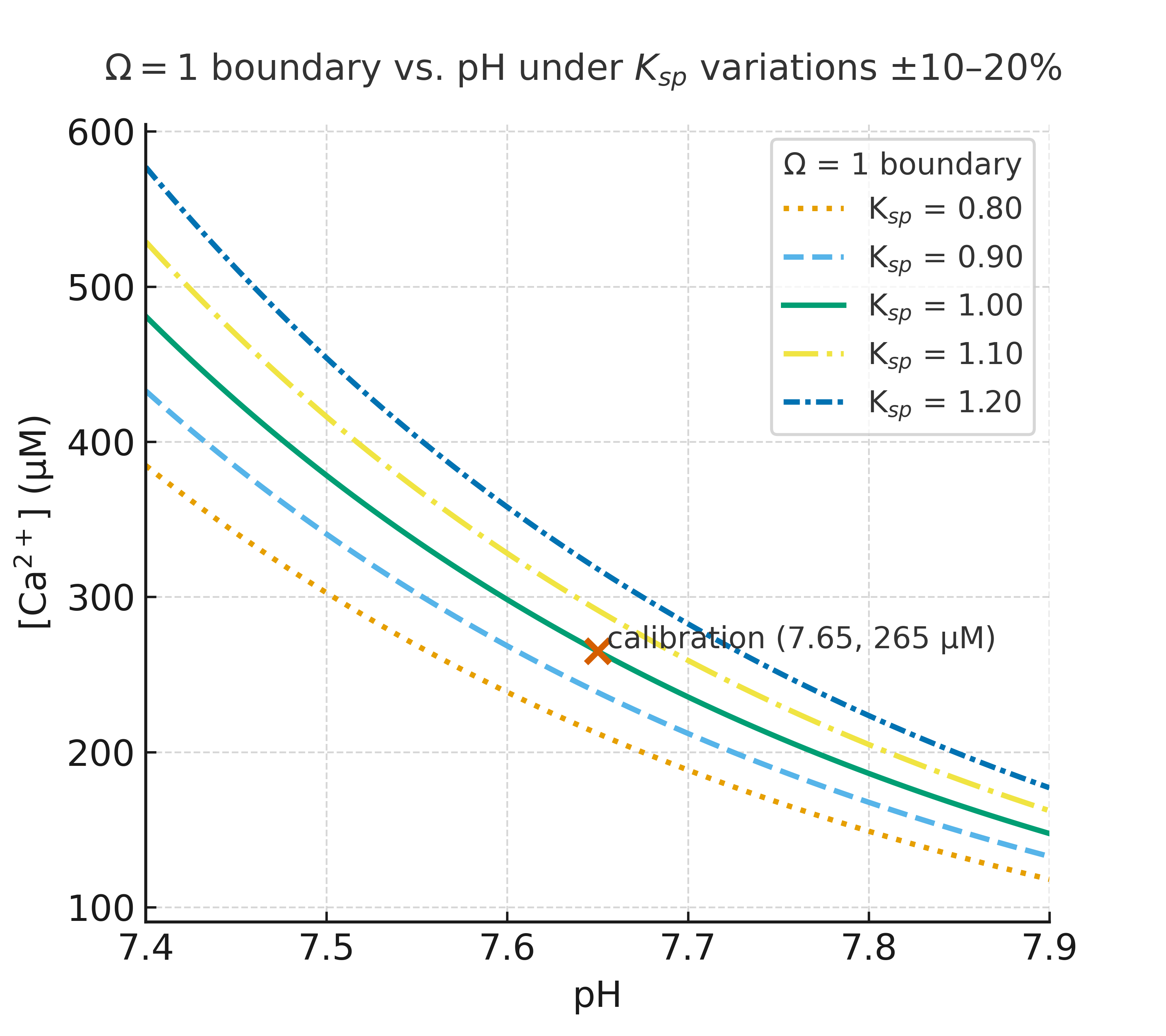

Supplement: Supplementary Figure S3 — Ω = 1 boundary as a function of pH under Ksp variations (±10–20%) at ~37 °C and I = 0.20 M. Curves show Ksp × {0.80, 0.90, 1.00, 1.10, 1.20}; the calibration point ( × ) denotes Ω = 1 at pH 7.65 and [Ca2+] = 265μM. Increasing Ks shifts the boundary upward (higher [Ca2+] required to maintain Ω = 1), while the qualitative topology is preserved. [file Image_3.tif]

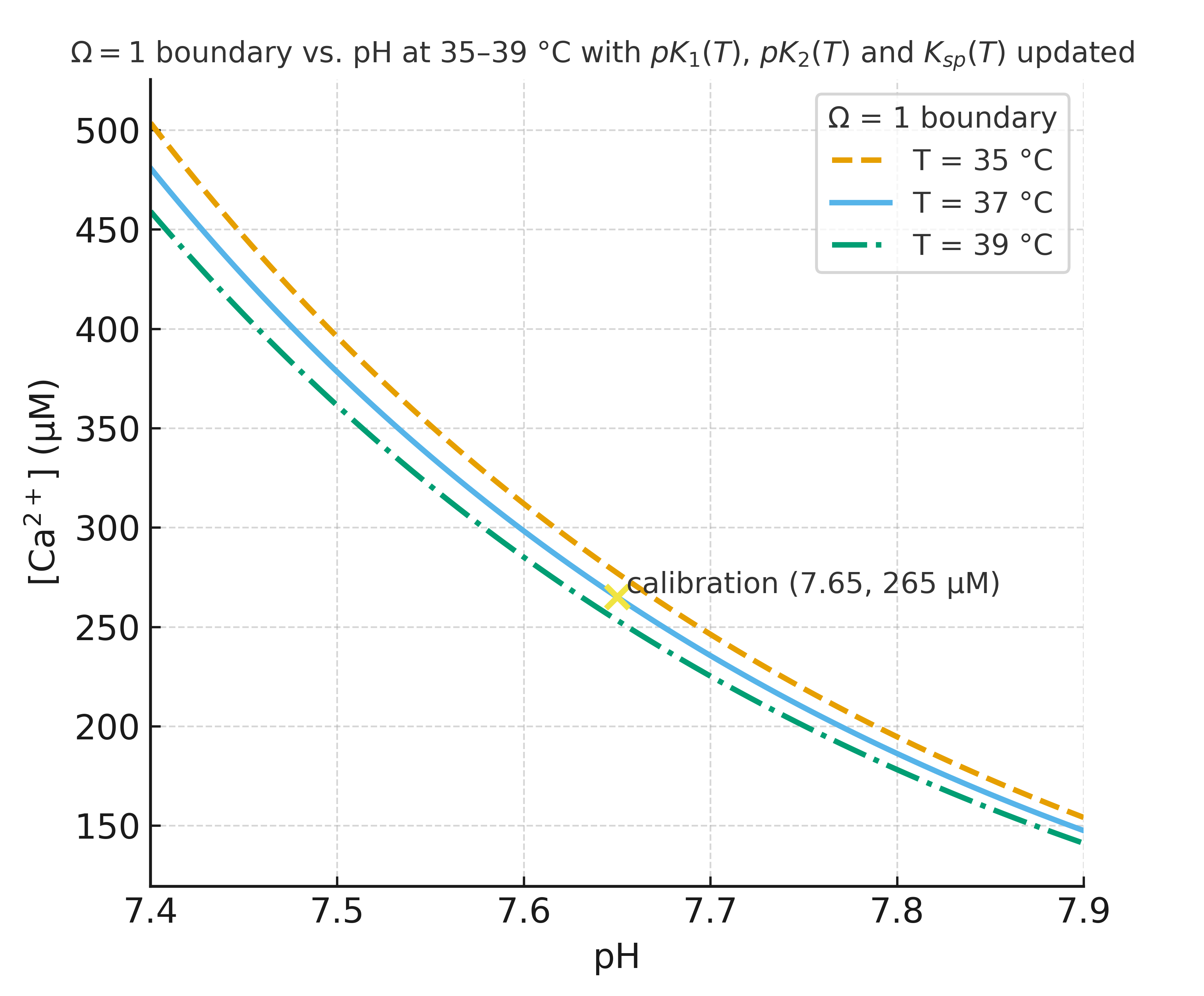

Supplement: Supplementary Figure S4 — Ω = 1 boundary vs. pH at 35–39 °C, updating pK1(T), pK2(T) and Ksp(T) (illustrative linearized adjustments) with I = 0.20 M. The calibration point (×) is set at 37 °C. Within this clinical range, higher temperature slightly lowers the required [Ca2+] to maintain Ω = 1, with the qualitative structure retained. [file Image_4.tif]

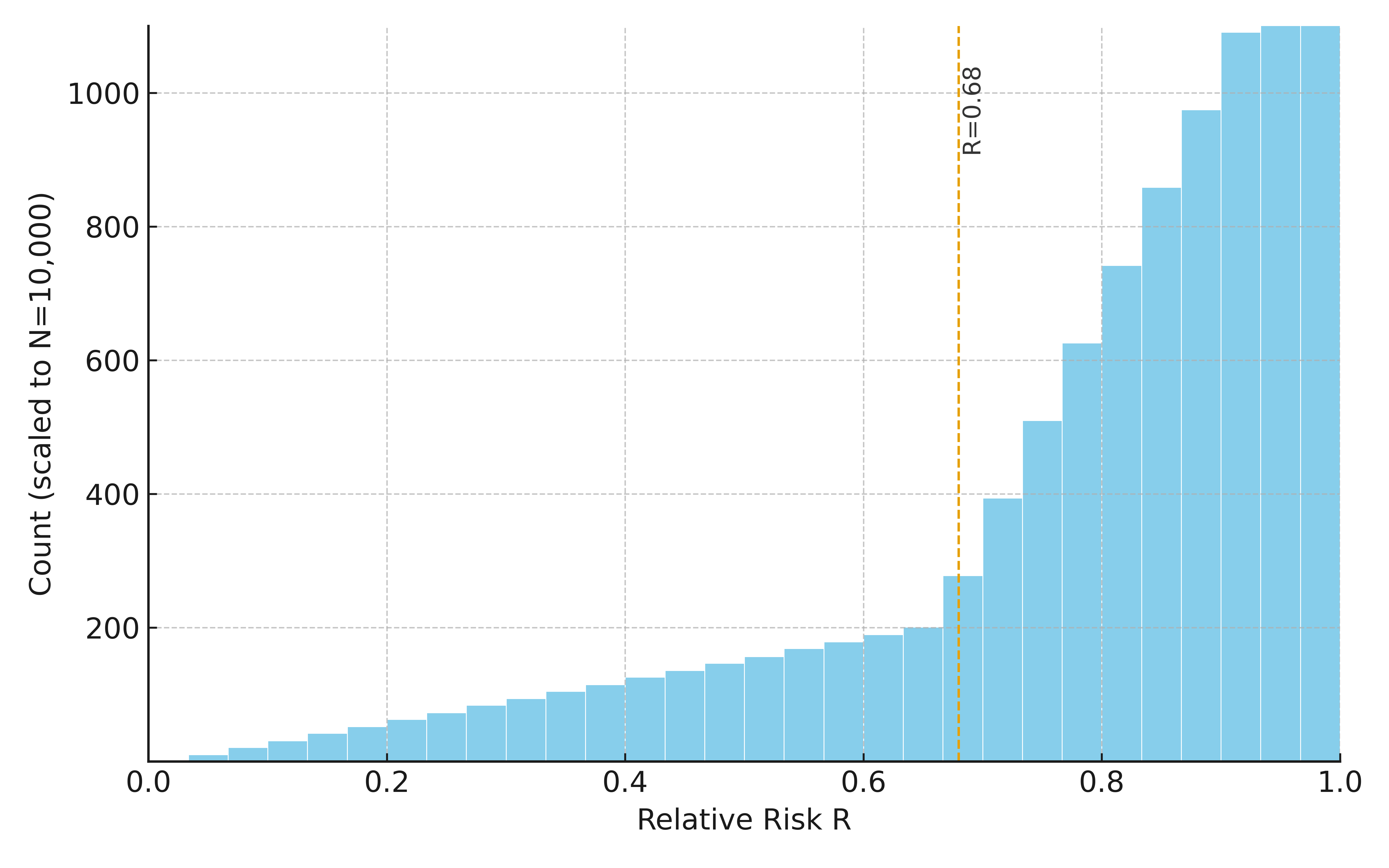

Supplement: Supplementary file 13 [file Data_Sheet_2.zip › outputs/Figure2_equal30_monoinc80_edgewhite_R068.tif]

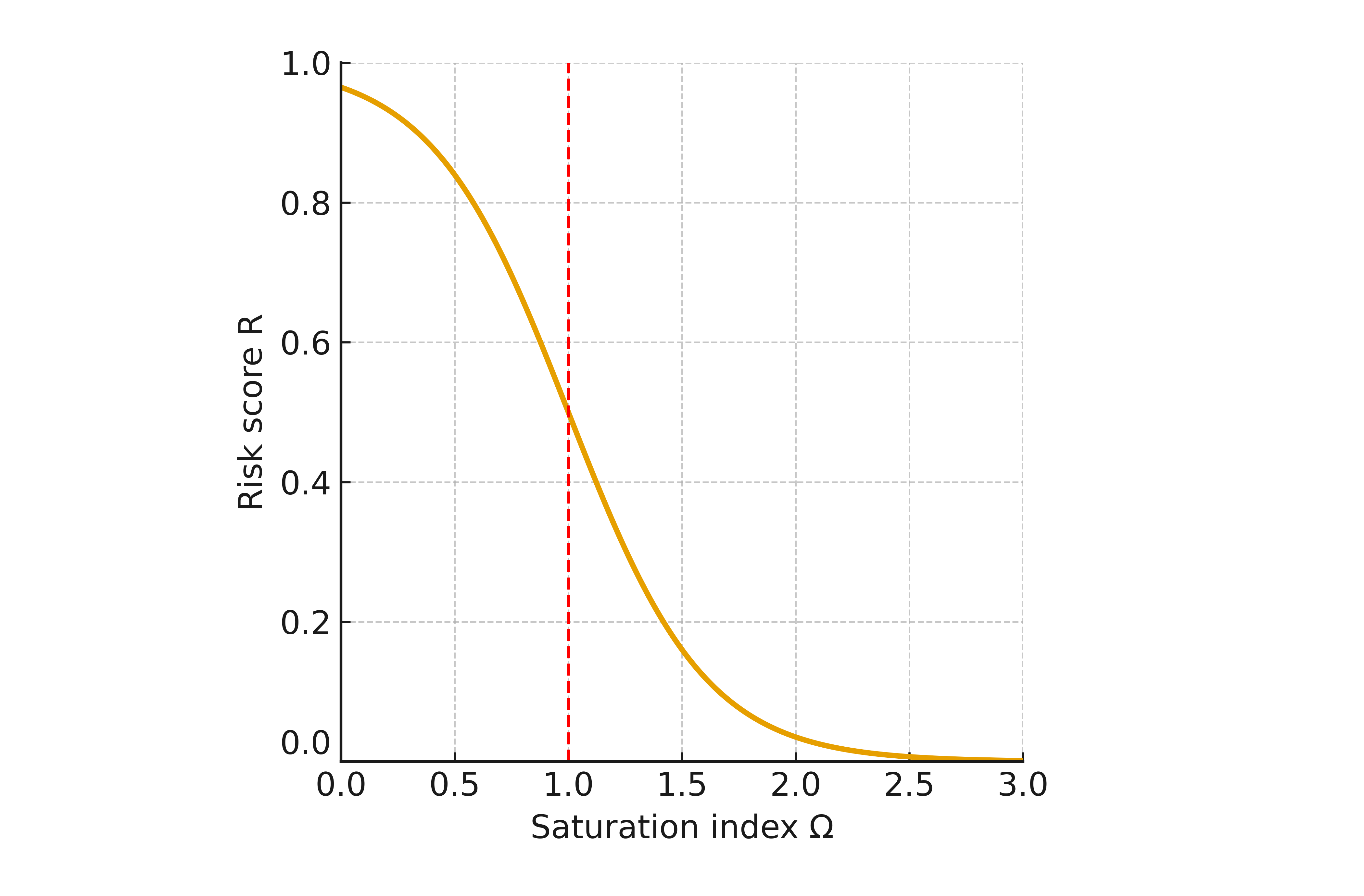

Supplement: Supplementary file 14 [file Data_Sheet_3.zip › outputs/Figure3_Omega_vs_R_x_to_3_clean_labels_redline_axisHalf_xticks05_y0labelshift03.tif]
